# Left side jejunal diverticulitis: US and CT imaging findings

**DOI:** 10.1016/j.radcr.2024.04.003

**Published:** 2024-04-20

**Authors:** Rosita Comune, Carlo Liguori, Francesco Guida, Diletta Cozzi, Riccardo Ferrari, Claudio Giardina, Francesca Iacobellis, Michele Galluzzo, Michele Tonerini, Stefania Tamburrini

**Affiliations:** a Division of Radiology, "Università degli Studi della Campania Luigi Vanvitelli", Naples, Italy; b Department of Radiology, Ospedale del Mare-ASL NA1 Centro, Naples, Italy; c Department of General and Emergency Surgery, Ospedale del Mare, ASL NA1 Centro, Naples, Italy; d Department of Emergency Radiology, Careggi University Hospital, Florence, Italy; e Department of Emergency Radiology, San Camillo Forlanini Hospital, Rome, Italy; f Department of Radiology, ASP of Messina-Hospital of Taormina, Messina, Italy; g Department of General and Emergency Radiology, “Antonio Cardarelli” Hospital, Via A. Cardarelli 9, Napoli, Italy; h Department of Emergency Radiology, Cisanello Hospital, Via Cisanello, Pisa, Italy

**Keywords:** Jejunal diverticulitis, Small bowel diverticulitis, Fecalized diverticulum, CT diagnosis of diverticulitis, Signs of acute diverticulitis

## Abstract

Small bowel jejunoileal diverticulosis is an uncommon and usually asymptomatic condition. Complications may occur such as acute diverticulitis including infection or perforation, bleeding, small bowel obstruction and volvulus. Herein we report a case of a 76 years-old woman with acute left side abdominal pain and tenderness. A clinical suspected diagnosis of colonic diverticulitis was formulated. She underwent Ultrasound that revealed a collapsed small bowel loop with a large sac-like out-pouching lesion with mixed content (fluid and pockets of air) associated to hyperechogenicity of perilesional fat. Because of the atypical US findings, the patient underwent abdominopelvic CT that confirmed that the large sac-like out-pouching was a jejunal inflamed diverticulum. The patient underwent emergency surgery. Radiologist should be aware of imaging findings of jejunoileal diverticulitis in order to achieve a prompt diagnosis.

## Introduction

Acquired jejunoileal non-Meckel diverticula are rare compared to large bowel diverticulosis and they occur in 0.03%-1% of general population [1], [2], [3]. This low frequency reflects the fact that these segments of small bowel are less frequently visualized during routine endoscopic examinations and diagnosis is made by imaging studies as incidental finding [4]. Jejunoileal non-Meckel diverticula are more common in males and their prevalence increases with age, peaking at the sixth and seventh decades [2,^5^,6]. The jejunum is more frequently involved (80%). They are often multiple except in the terminal ileum [2,^7^,8] and tend to be larger and higher in number proximally and smaller and fewer when progressing distally in the small bowel [2,^6^,[9], [10], [11]. Small bowel diverticula are usually asymptomatic, and around 30% of patients are symptomatic, while only 10% of patients proceed to develop complications including, obstruction, fistula formation, peritonitis, lower gastrointestinal bleeding, and perforation [2,^3^,^10^,[12], [13], [14]. Patients may present diffuse abdominal pain, fever, nausea, vomiting, abdominal bloating, and diarrhea; the prevalence of symptoms varies among different studies, confirming the nonspecific clinical presentation [1,^3^,^5^,15]. This variability depends also by the location of diverticula that may generate different clinical picture. Complications unfortunately carry a high mortality rate in case of perforation (25%-50%) [3,^16^–19], and the non-specific clinical presentation may negatively influence the diagnosis in early stages and delay the diagnosis.

We report a case of a 76 years-old woman of left side jejunum diverticulitis diagnosed at US and CT.

## Case presentation

We report a case of a 76-year-old woman who presented to our emergency department complaining of acute abdominal pain which she referred was gradual in onset, dragging in character, and nonradiating. Her past medical history included coronary artery disease treated with PCI, thyroidectomy (12 years ago due to thyroid goiter). She had natural deliveries and no history of abdominal surgeries. The physical examination showed pain localized in the left lower quadrant with peritonism and involuntary guarding during deep palpation.

Cardiorespiratory auscultation was normal. She was afebrile and hemodynamically stable.

**Fig. 1:**
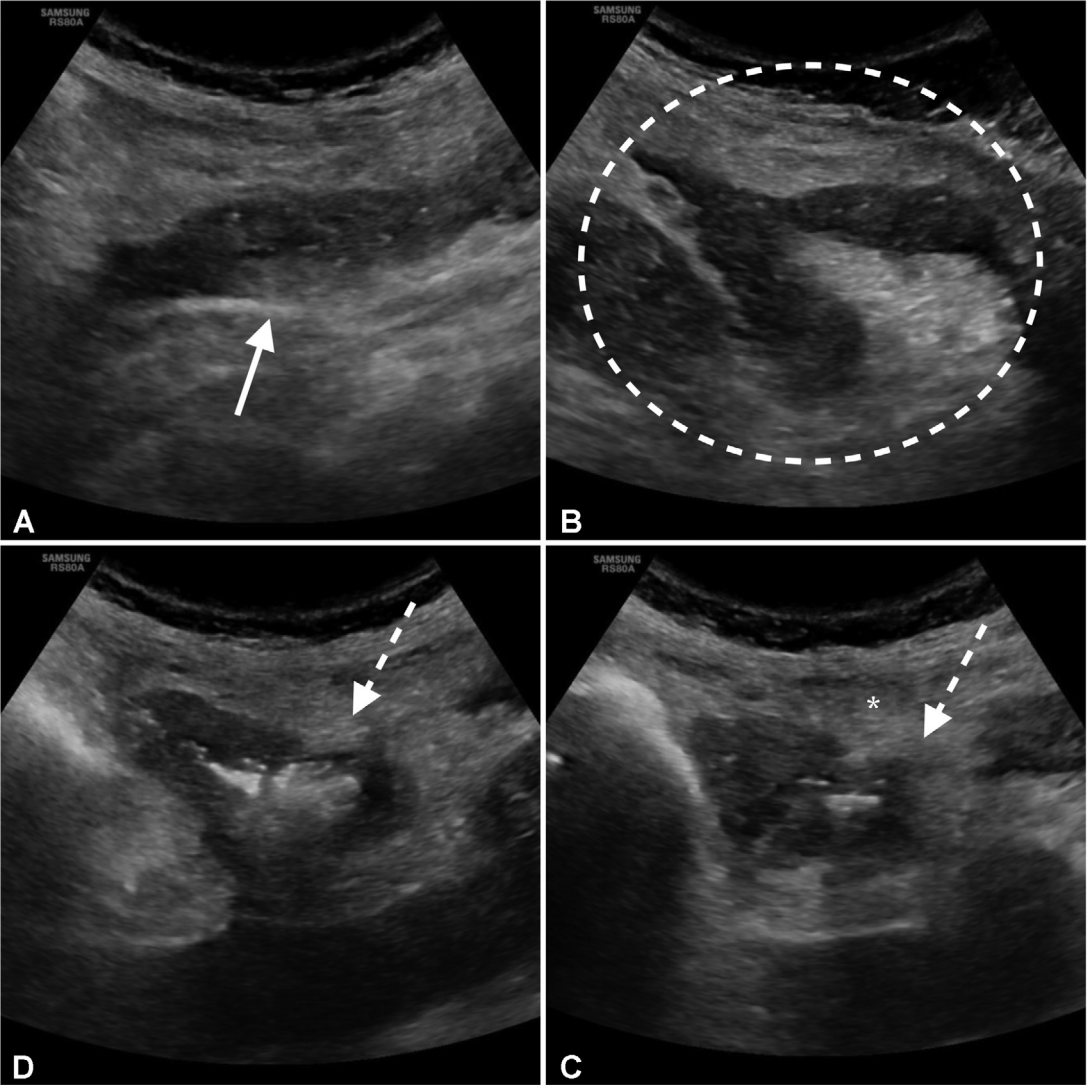
(A-D) Ultrasound examination with convex probe. At the left lumbar region, a small bowel loop with eccentric thickening was appreciated with ill-defined margin on the mesenteric side (with arrow) (A) Adjacent small bowel loops were collapsed and angled (dashed circle) (B) An outpouching lesion with thickened walls and mixed content was detected in connection with a bowel pool (dashed arrow) (longitudinal C, axial D). The adjacent mesenteric fat was inhomogeneous, thick, and hyperechogenic (asterix).

**Fig. 2:**
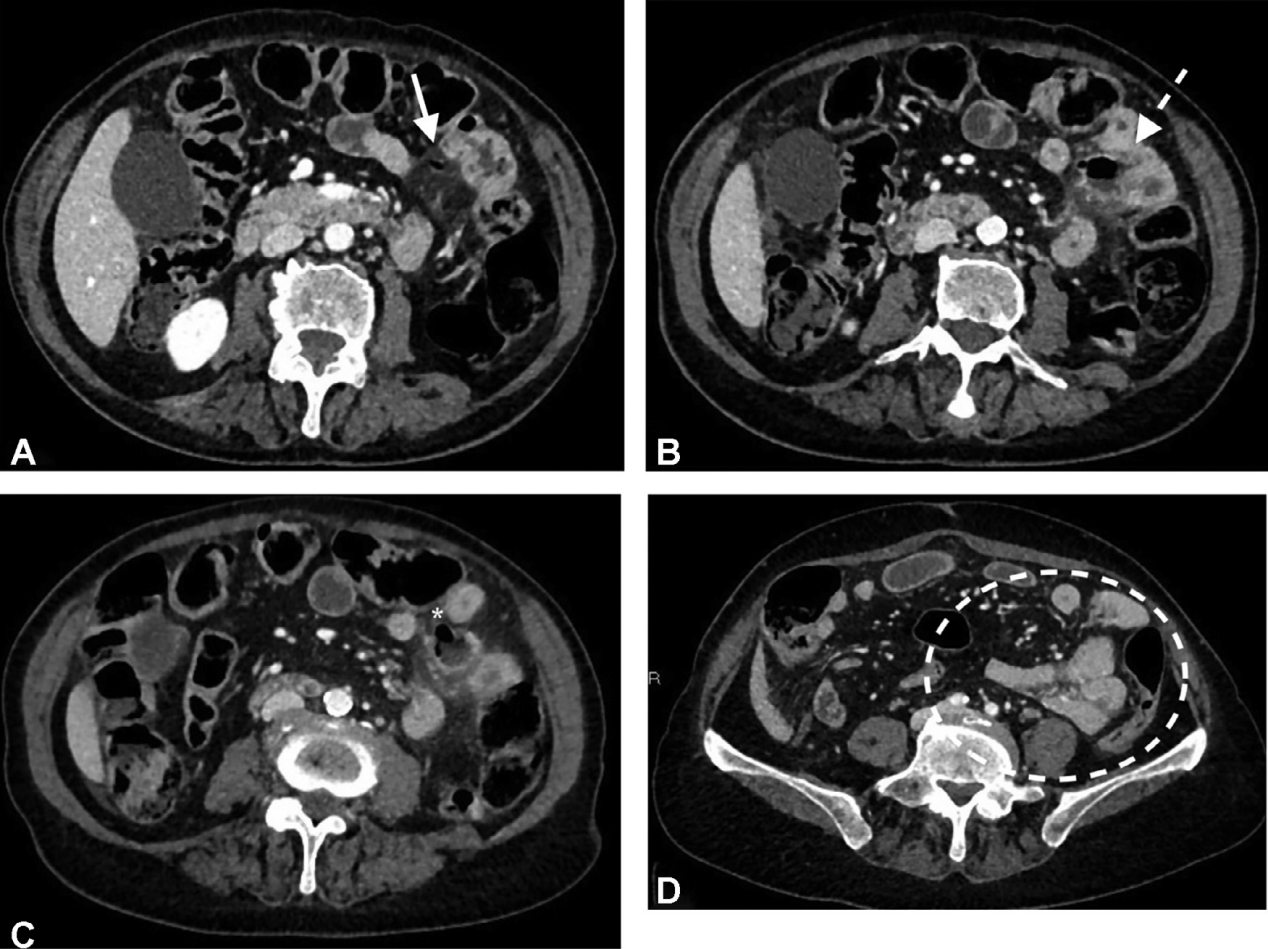
(A-D) Axial CT contrast images (parenchymal phase). A millimetric extraluminal air bubble is detected strictly close to the mesenteric side of the jejunal loop (white arrow) (A) The out-pouching lesion is detected in connection with the jejunum. The diverticular sac is air filled with thick walls (dashed arrow) (B) On the anterior side of the diverticular sac the wall is not appreciable (asterix) (C) The adjacent bowel presents eccentric thickened and hyperemic walls. Other jejunal loops are collapsed and angled resembling identically the US findings (dashed circle) (D) Stranding of peri-diverticular sac is visible on the mesenteric side.

**Fig. 3:**
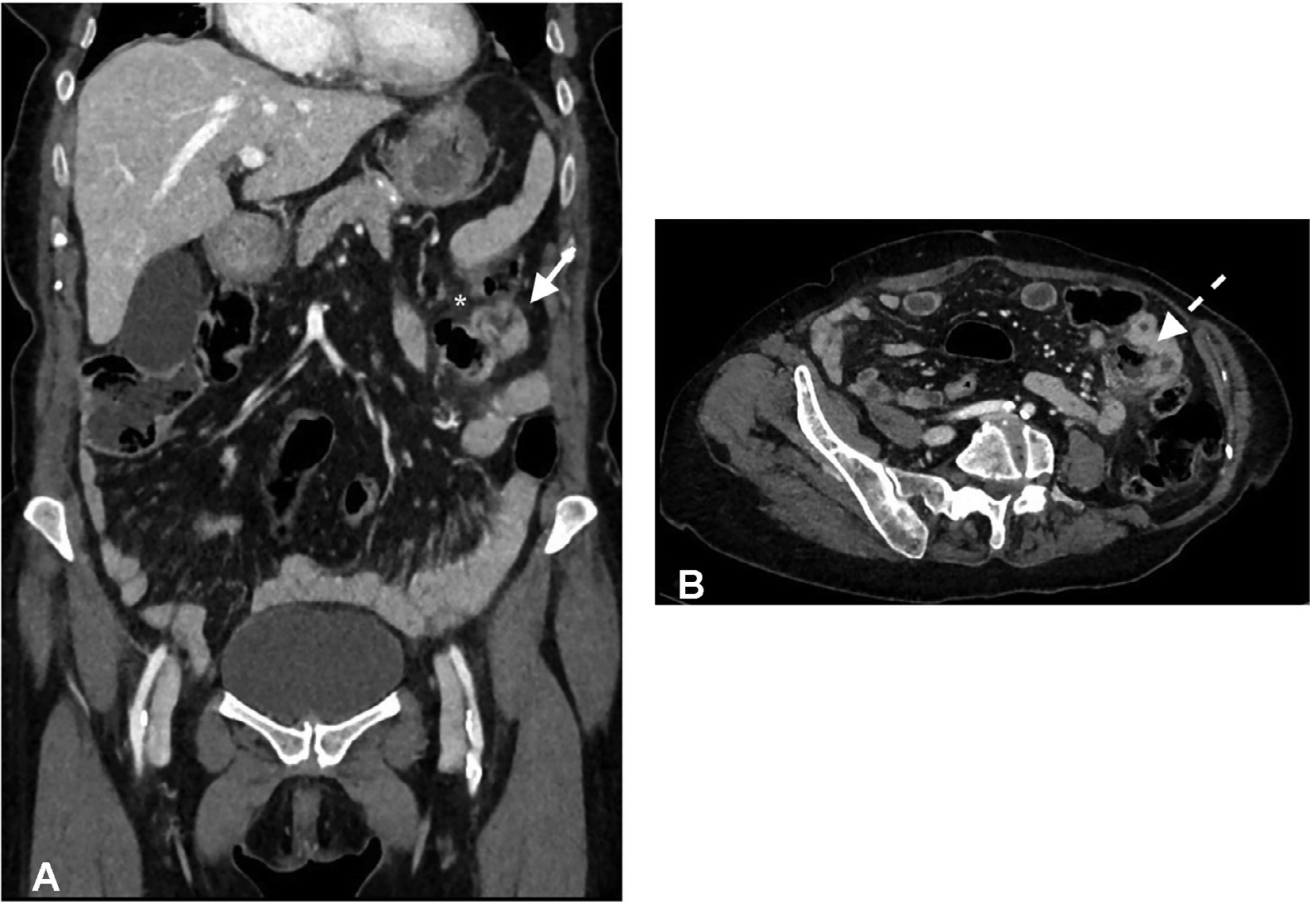
(A and B) Coronal and para-axial CT contrast images (parenchymal phase). The thickened jejunum loop is distinctly evident with the out-pouching diverticula sac on the mesenteric side (white arrow) with perilesional fat stranding (asterix) (A). On para-axial image, the diverticular neck is clearly visible (dashed arrow) (B).

Upon admission, complete blood count and metabolic panel revealed increase of white blood cells (14,25 10^3^/mm^3^, n.v. 4.2-10.5) neutrophils (13,20 10^3^/mm^3^, n.v 40,0-75), d-dymer (908 microg/L, n.v. 10-500). The rest of metabolic panel and blood count was unremarkable, and CPR was in the range (0.38 mg/dL, n.v.0.0-0.5 mg/dL). The Ultrasound exam was performed with the convex probe for the thick adipose panniculus of the patient. No free fluid or fluid collections were detected. No ultrasound signs of nephrolithiasis or cholestasis were evident neither abnormal distension nor kinesis of small bowel. Adjacent bowl loops were collapsed and angled. Carefully US examination of left lower quadrant, guided by the patient's symptomatology, allowed to identify a thickened and collapsed small bowel loop with an irregular out-pouching lesion with thickened walls and mixed content (fluids and hyperechogenic spot of air). Prelesional fat was hyperechogenic (Figs.1A-D). The cause of abdominal pain was suspected at US to be the anomalous out pouching lesion of a small bowel loop and CT with intravenous contrast was performed. The patient underwent MDCT with intravenous contrast (1.0–1.5 mL/kg injected at 3.5 mL/s, followed by 50 mL of a saline bolus injection). A CT multiphase protocol was acquired (noncontrast, arterial, and parenchymal). At CT, a jejunum loop with eccentric wall thickening was appreciable in the left lumbar region with an outpouching lesion on the mesenteric side (Figs. 2A-D). The diverticulum neck was visualized on axial and more evident on MPR reconstruction (Fig. 3A-B). Stranding of adjacent mesenteric fat was evident only on mesenteric side. The diverticulum had mixed content (fluid and gas), thickened wall with irregular margin and a focal defect. A millimetric air bubble was appreciable outside the diverticular sac within the mesenteric fold (Figs. 2A-D). A final diagnosis of complicated jejunal diverticulitis with sealed perforation was formulated and the patient underwent emergency surgery.

At laparotomy a single diverticulum on mesenteric side of the jejunum was found. The diverticulum wall was ischemic with focal sealed perforation and a diverticulectomy using a mechanical device (Echelon 45 mm) was performed. The suture was reinforced by surgical glue. The postoperative course was uneventful, oral intake was started in fourth postoperative day and the patient was discharged on the seventh postoperative day. Histopathological examination of the resected diverticulum revealed inflammatory changes with focal perforation.

## Discussion

Acquired diverticula are false diverticula or pseudodiverticula as they lack muscular layer present instead in true diverticulum. They may be primary or secondary [7,^10^,20]. Secondary acquired diverticula develop as consequence of abdominal operations, sclerodermia, tuberculosis, and Crohn's disease [2,^7^,19]. They are usually multiple and occur at the mesenteric border of small bowel by herniation of the mucosa and submucosa [21]; their size varies from a few millimeters to more than 10 cm.

Association with colonic diverticula has been reported in up to 91% of cases [3,^7^,^12^,^19^,^22^–24]. Pathogenesis of small bowel diverticula may be the same of large bowel diverticula: intestinal dyskinesia caused by abnormalities of the smooth muscle and myenteric plexus determine increase of segmental intraluminal pressure [25] causing a mucosal herniation through the weakest mesenteric site of the bowel wall along the area where the vasa recta and nerves penetrate the mesentery area [3,^26^,27]. Complicated jejunoileal diverticulitis is rare [19] and clinically it can mimic many other surgical and nonsurgical causes of acute non traumatic abdominal pain depending by the tract involved (jejunum or ileum or terminal ileum) [3,^5^,^16^,18].

US and CT findings of jejunoileal diverticulitis have been described and imaging findings resemble the US and CT pattern of colonic diverticulitis [2,^3^,^6^,12]. Ultrasound is the first line imaging to screen patients with nonspecific abdominal pain [3,28]. In ER settings, abdomen should be examined completely to avoid satisfaction of search (SOS) errors [29]. Accuracy of US in colonic diverticulitis has been reported with extreme variability (sensitivity of 77% to 98% and a specificity of 80% to 99%) [30,31] because bowel ultrasound accuracy in ER settings require high level of expertise of the operator. The exam should be guided by clinical symptoms and the area where the pain is reported by the patient should be carefully explored. At ultrasound, the loop involved appears thickened with a sac like outpouching lesion with hypoechoic wall and inhomogeneous content. Signs of acute diverticulitis are hyperechogenic perilesional fat, fluid filled collection with or without hyperechogenic spot of air [2,12]. Although jejunoileal diverticulitis can be detected at US, ultrasound remains less sensitive and cannot replace CT [3,^19^,32]. CT scan is considered the gold standard for jejunoileal diverticulitis although its accuracy is reported from 35% to 65% [5,6] because CT diagnosis of jejunoileal diverticulitis is challenging. Fintelman et al. [6] reported that CT accuracy's decreases in case of dilated small bowel because it is more difficult to recognize the inflamed diverticulum because given that effacement of folds and thinning of the wall in dilated small-bowel loops make more difficult to recognize jejunal diverticula [6]. De Simone et al. [5] reported that CT accuracy varies according to the complication occurred, with a diagnostic rate of 80% in case of perforation and 36.7% in case of diverticulitis [3,5]. In this scenario, CT findings of jejunoileal diverticulitis should be deeply analyzed.

Jejunoileal diverticula appear as round or ovoid, fluid—, feces—, or air—out pouching structures adjacent to the mesenteric side of the small bowel, with barely discernible wall and nonrecognizable small-bowel folds [6]. When inflammation occurred, the wall becomes appreciable due to peridiverticular edema and thickening. Eccentric wall thickening of the adjacent bowel loop can be detected in case of severe inflammation [33]. The epicenter of inflammation is the mesenteric border where diverticula originate [34], and the anti-mesenteric side is usually involved by inflammation only in severe cases. Multiplanar CT Reconstruction are extremely helpful in determining the epicenter of inflammation along the mesenteric bowel border adjacent to the diverticulum, helping radiologists to localize the disease and to identify the diverticular neck [6,^19^,35]. The “fecalized diverticulum”, defined when fecalized content is seen within the diverticula, was identified by Chapman et al. [19] in 51% of patients with jejunoileal diverticulitis helping in identifying the culprit diverticulum. When perforation occurs extraluminal free air, mesenteric inflammation, and/or localized fluid collections can be detected [3,^16^,^17^,^19^,36]. Because small bowel diverticula are often multiple, except for the terminal ileum, careful inspection should be done along other the small bowel [2,^12^,13]. Conservative management is the initial treatment option for patient with no life-threatening complications [17,37]. However, surgery is the preferred treatment option, especially in the presence of complications [3,^5^,^23^,38]. In our case, the out-pouching diverticular lesion was located anteriorly making possible the visualization at US that is usually not possible if the diverticulum is located posteriorly because of the barrier created by intraluminal gas [3].

At CT, the imaging features of jejunum diverticulitis were clearly evident on axial and MPR reconstruction. The outpouching sac was located on the mesenteric side and *valvulae conniventes* were not appreciable within. The sac was connected (diverticulum neck) to the adjacent jejunal loop that presented eccentric thickening and parietal hyperemia. The mesenteric fat stranding was appreciable only on the mesenteric side with the diverticulum as epicenter. These findings allowed a confident diagnosis of jejunum diverticulitis.

## Conclusion

Small bowel diverticulitis is a rare pathology with different clinical presentation that can mimic many pathologies. At US the inflamed diverticulum may be appreciable if located anteriorly but the imaging of choice for the diagnosis is CT. CT is not highly accurate, although it represents the gold standard for the diagnosis of jejunoileal diverticulitis. Many factors may influence CT diagnosis negatively. First of all the presence of adjacent dilated small bowel loop that may make diverticulum recognition difficult. Strangely CT diagnostic accuracy depends by the type of complications being higher in case of perforation and significantly lower in case of non-complicated diverticulitis. Carefully inspections of images in axial and multiplanar reconstruction may help to identify the diverticulum because its identification is the mainstay of the diagnosis. At CT, the diverticulum appears as an outpouching lesion with thickened and hyperemic walls containing fluid and air bubbles or fecaloid material (fecalized diverticulum). In case of perforation, the diverticulum wall can be focally interrupted and extraluminal air bubbles or abscesses can be detected. Another important CT finding is the inflammation of the mesenteric fat that is localized typically along the mesenteric border. Radiologist should be aware of the characteristic and distribution of jejunoileal diverticulitis and of CT imaging features in order to achieve and early diagnosis.

## Patient consent

Written informed consent was obtained from the patient for publication of this case report and accompanying images. A copy of the written consent is available for review by the Editor-in-Chief of this journal on request.
